# Nitrosamines and Polycyclic Aromatic Hydrocarbons in Smoke-Cured Bacon (Larou) of Artisanal and Industrial Origin

**DOI:** 10.3390/foods10112830

**Published:** 2021-11-17

**Authors:** Lei Chen, Rui Liu, Mangang Wu, Hai Yu, Qingfeng Ge, Wangang Zhang

**Affiliations:** 1 College of Food Science and Engineering, Yangzhou University, Industrial Engineering Center for Huaiyang Cuisine of Jiangsu Province, Yangzhou 225127, China; leichen_yzu_edu@163.com (L.C.); ruiliu@yzu.edu.cn (R.L.); mgwu@yzu.edu.cn (M.W.); yuhai@yzu.edu.cn (H.Y.); 2 College of Food Science and Technology, Nanjing Agricultural University, Nanjing 210095, China

**Keywords:** Larou, nitrosamines, polycyclic aromatic hydrocarbons, artisanal origin, industrial source

## Abstract

The aim of this study was to compare the nitrosamines (NAs) and polycyclic aromatic hydrocarbons (PAHs) of Chinese smoke-cured bacon (Larou) of artisanal and industrial origin. The results showed that the average pH and Aw values of family-made Larou products were lower than those of industrial Larou, which was opposite to the TBARS level. The contents of residual nitrite and PAH4 in two artisanal Larou were significantly higher than those of the other groups (*p* < 0.05). The highest NA content (10.78 μg/kg) was found in family-made Larou. A correlation analysis indicated that the relationships between residual nitrite contents and total PAH8 contents (τ = 0.692, *p* < 0.01) and total NAs contents (τ = 0.805, *p* < 0.01) were characterized with a positive correlation. A principal component analysis indicated that the Larou from the industrial sources had similar safety attributes, and was more stable than the Larou processed in an artisanal manner. Our data suggest that Larou produced in industrial conditions is suitable for consumption.

## 1. Introduction

Larou, a traditional Chinese smoke-cured bacon, has a history of 3000 years and is processed by families in the cold lunar month of December before the Chinese New Year. Generally, it is prevalent in the south of China, especially in Sichuan, Hunan and Guangdong provinces. Nowadays, Larou is commercialized and industrially produced with a great economic value, having reached a market value of 67 billion ¥ in 2016. Larou is usually prepared from longissimus thoracis, streaky pork or pork leg by salting, smoking or dry-curing and ripening [1]. Salt is usually added during pickling, while ingredients including paprika, liquor and spirits or other spices are chosen to be optionally added. The production of handcrafted Larou takes place mainly in smokehouses of family farms. According to traditional processes, the composition and the amount of pickled powder are variable among regions and families, generating different flavors of Larou products. The time of salting in the artisanal mode ranges from 2 to 7 days (d) depending on the temperature and humidity of the cold weather in different places. In contrast, the Larou under industrial conditions undergoes processes such as brine injection, which shortens the salting time to 48–72 h at 4–6 °C by automatic control.

Smoking is of great importance for extending Larou’s shelf-life, due to the formation of various compounds which contain antimicrobials and antioxidants [2]. Fruitwood, pinewood and firewood are ideal and economical heat sources for smoking, which contribute to the appealing color, aroma and flavor of Larou. However, due to the incomplete combustion and pyrolysis processes applied to the wood, polycyclic aromatic hydrocarbons (PAHs) are formed and adsorbed on the surface of Larou, and accompanied by health concerns [3]. According to the National Standard of the People’s Republic of China [4], the maximum allowed content of benzo(a)pyrene (BaP) for smoked, grilled and barbecued meat products is 5 μg/kg. The technique of smoking, the choice of wood, the duration of smoke exposure and the raw meat material are critical factors that affect the occurrence of PAHs during the smoking process [5]. In contrast to the farm’s dry wood smoking, liquid smoke and an automatic smoking oven have been used in industrial meat products as an alternative process [6]. As far as this process is concerned, hazardous compounds in artisanal and industrial Chinese Larou are rarely investigated.

Sodium nitrite has been widely used for preservation in the production of traditional Chinese Larou for decades, since it can develop its characteristic red color and flavors, and inhibit lipid oxidation and microorganisms [1,7]. However, nitrite is considered as the main source of nitrosating agents, and the positive correlation between nitrite content and nitrosamine (NA) formation in meat products has been reported [8,9]. Moreover, NAs are usually also formed in meat products by the reduction of nitrates to nitrites and the subsequent reaction with amines from the proteolysis of meat proteins [10]. Many NAs, including N-nitrosodimethylamine (NDMA), N-nitrosodiethylamine (NDEA), N-nitrosodibutylamine (NDBA), N-nitrosopiperidine (NPIP), N-nitrosopyrrolidine (NPYR) and N-nitrosomorpholine (NMOR), have been classified as possible carcinogens by the International Agency for Research on Cancer (IARC), while they are normally detected in smoked meat products. Commercial meat products of Estonia and Denmark have been reported to have low nitrosamine contents when produced under good manufacturing practices (GMP) [8,11,12]. Tbran, Cordi, Becze, Dan and Mihaiu [13] found total PAHs in the traditionally smoked bacon, and the lowest PAHs in industrially produced pork sausage. Moreover, studies were conducted to investigate the curing (i.e., the concentration and substitutes of salts) and drying technologies to reduce the accumulation of NAs and PAHs in bacon [14,15]. However, there is still no knowledge on the potential risks of NA and PAH contents in Larou from farmhouses and commercial retail. Therefore, the aim of this research was to investigate the differences in the PAH and NA contents of Larou from farmhouses and industrial manufacturers. Moreover, the contents of TBARS and residual nitrites were used as indicators to provide consumers with knowledge on how to reasonably choose Larou products.

## 2. Materials and Methods

### 2.1. Sample Collection

Three kinds of bulk family-made Larou samples were purchased from local farmers in Shaoyang, Changsha and Zhangjiajie, in Hunan Province, designated as SY, CS and ZJ, respectively. The other three vacuum-packed factory-made Larou samples were obtained from Hunan Guanghui Food Co., Ltd. (Changsha, China, named YPT), Yushanghuang Meat Products Factory, (Liuyang, China, named YSH), and Hunan Tangrenshen Group Co., Ltd. (Zhuzhou, China, named TRS), respectively. The raw meat of Larou from all sources was pork belly. The thickness of Larou for the SY, CS, ZJ, YPT, YSH and TRS groups was 5, 8, 6, 8, 3 and 6 cm, respectively. Three production batches for each type of Larou sample (artisanal and industrial) were considered, and three Larou products from each batch were collected. Each Larou sample (500 g) was stored at −20 °C for further analysis.

### 2.2. pH Measurement

A digital pH-meter (Mettler Toledo, HM-5S; TOA Electric Industrial Co. Ltd., Tokyo, Japan) was used to measure the pH value, which was determined according to Karlsson and Rosenvold [16], with some modifications. Standard buffers of pH 4.01 and 6.86 (Thermo Fisher Scientific (China) Co., Ltd., Shanghai, China) were used to calibrate the pH meter at 20 °C. The Larou samples (10 g) were homogenized with 50 mL distilled water for 10 s twice at 25,000 rpm on ice, with a 15 s interval between bursts. After equilibration at 20 °C, the pH was recorded.

### 2.3. Determination of Water Activity (Aw)

Aw was analyzed by an Aw meter (Novasina AG, Novasina, Switzerland). A saturated NaCl solution was used to calibrate the Aw meter. Larou (3 g) was minced and placed in the measuring chamber for 10 min at 25 °C. The Aw value was recorded after the measurement had been completed.

### 2.4. Determination of Residual Sodium Nitrite

The content of residual sodium nitrite was detected by a colorimetric method according to the National Standard of the People’s Republic of China [17].

### 2.5. TBARS Detection

The TBARS of each Larou was measured according to the method of Zhang et al. [18], with some modifications. Minced Larou samples (5 g) were homogenized with 25 mL of a 7.5% (*m*/*v*) trichloroacetic acid solution and a 0.1% (*m*/*v*) EDTA for 10 s twice at 25,000 rpm on ice. After the homogenate was filtered through filter paper (8 μm, Chengdu Dianrui Experimental Instrument Co., Ltd., Chengdu, China), 5 mL of a 0.02 M thiobarbituric acid solution was added to the filtrate. The sample was mixed and incubated in a boiling water bath for 40 min. After the mixture cooled in water at 4 °C, the sample was centrifuged at 2000× *g* for 5 min at 4 °C and then mixed with 5 mL chloroform for 20 min. The absorbance of the resulting upper layer was measured at 532 nm and 600 nm by using an automatic microplate reader (TECAN M200 Pro, Tecan Trading AG, Switzerland). The TBARS values were calculated using the following formula: TBARS (mg/1000 g) = (A532−A600) × 4.68 × (10/5). The values were expressed as mg of malondialdehyde (MDA) per kg of Larou.

### 2.6. Analysis of PAHs

The extraction of PAHs was performed according to the method described by Wang et al. [19]. Briefly, 3 g of a lyophilized Larou sample was placed into a glass centrifuge tube (50 mL), and 20 mL of acetonitrile and 10 mL of n-hexane (Macklin Inc., Shanghai, China) dissolved in 100% acetonitrile were added. The samples were shaken for 30 s and then extracted with ultrasonic treatment at a power of 600 W and 40 kHz for 30 min at 40 °C. After homogenization, the samples were centrifuged at 10,000× *g* for 5 min, and the acetonitrile layer was collected. The extraction process was repeated once. The solvents of the acetonitrile layer were combined and evaporated using a rotatory evaporator (Shanghai Yaote Instrument Technology Co., Ltd., Shanghai, China) at 35 °C. The residue was resuspended in 6 mL of n-hexane and loaded onto a Silica cartridge (20 mL, Waters Corporation, Milford, MA, USA), which was equilibrated with 20 mL of 100% dichloromethane and conditioned with 20 mL n-hexane. The cartridge was eluted with 25 mL of n-hexane/dichloromethane (50:50, *v*/*v*). All the eluent was collected and then evaporated to be dried by a nitrogen stream (Shanghai Jingsheng Scientific Instrument Co., Ltd., Shanghai, China). The concentrate was dissolved in 100 μL of acetonitrile/methanol (90:10, *v*/*v*) and filtered through a 0.22-μm membrane filter before analysis.

The separation and quantification of PAHs were performed by ultra-performance liquid chromatography with diode array and fluorescence detection (UPLC-DAD/FLD) according to the method described by Viegas, Yebra-Pimentel, Martínez-Carballo, Simal-Gandara and Ferreira [20], with some modifications. A UPLC unit (Agilent Technologies, Inc., Santa Clara, CA, USA) equipped with a G4220B pump, a 10 μL loop G4226A auto-sampler, a G4212A diode array and a G1321B fluorescence detector was used. The separation of PAH was performed with a 10 cm × 3 mm × 3 μm particle size Supelcosil LC-PAH (Supelco, Bellefonte, PA, USA) at 30 °C. Acetonitrile (A) and ultrapure water (B) were used for the mobile phase at a flow rate 0.64 mL/min. The injection volume was 4 μL. The linear gradient was set as follows: 0–2 min, 50% A, 50% B; 2–8 min, 50–100% A, 50–0% B; 8–11.2 min, 100% A, 0% B; and 11.2–15 min 100–50% A, 0–50% B. The diode array detector was set to 254 nm. The excitation and emission wavelengths were 270 and 390 nm for benzo(a)anthracene (BaA) and chrysene (Ch); 260 and 430 nm for benzo(b)fluoranthene (BbF); 290 and 410 nm for benzo(k)fluoranthene (BkF), BaP, dibenzo(a,h)anthracene (DbA) and benzo-(g,h,i)perylene (BgP); and 293 nm and 498 nm for indeno(1,2,3-c,d)pyrene (IP), respectively. The compounds were identified by comparing their retention time to those of the reference standard mixture of PAHs. A quantitative determination was performed using an external calibration curve.

### 2.7. Analysis of NAs

Larou samples (5 g) containing internal standards (NDMA-d6 and NDPA-d14, Macklin Inc., Shanghai, China) were extracted with 10 mL extraction buffer containing 50% acetonitrile and 30% ammonium formate in a 50 mL tube. After vortex for 2 min and sonication (ultrasonic power at 600 W and 40 kHz) for 30 min at 25 °C, the mixture was centrifuged at 8000× *g* for 10 min at 4 °C. The samples were frozen at −20 °C for 16 h. Then, 5 mL of supernatants were quickly added into solid phase extraction cartridges, including 150 mg primary secondary amine, 150 mg carbon 18 and 900 mg magnesium sulfate (Agilent Technologies, Inc., Palo Alto, CA, USA). After vortex for 2 min, the mixture was centrifuged at 8000× *g* for 10 min at 4 °C. The elution was evaporated to 1 mL by a nitrogen stream and filtered through a 0.22 μm membrane filter prior to the GC-MS analysis. The separation of NAs was performed using an Agilent DB-5MS (30 m × 0.25 mm × 0.25 mm) fused-polyethylene glycol capillary column (Agilent Technologies, Palo Alto, CA, USA). The GC oven temperature was maintained at 38 °C for 4 min, and then increased to 83 °C by 8 °C/min, held for 4 min and lastly raised to 250 °C by 15 °C/min. The injection was performed at 250 °C, and the electronic pressure constant flow was set as 1 mL/min. Mass spectrometry was performed in the electron-ionization mode with 70 eV of ionization energy. The ion source temperature was 250 °C. All analyses were performed in the selected ion monitoring mode (SIM) of the MS in order to enhance the selectivity and sensitivity. The analytes were qualitatively determined by the internal standard and quantified using the external calibration.

### 2.8. Statistical Analysis

All analytical tests were carried out in triplicate, and the results were expressed as means ± standard deviation. The results were statistically analyzed by a one-way ANOVA. The comparison of the mean values was performed using Duncan’s test by SPSS for Windows version 16 (IBM Corporation, Armonk, NY, USA). The significance was considered as *p* < 0.05. The plots were made by using Origin for Windows version 10 (OriginLab Corporation, Northampton, MA, USA). A principal component analysis was performed using the statistical software SIMCA 14.1 (MKS Instruments AB Inc., Malmo, Sweden) setting nitrosamines, polycyclic aromatic hydrocarbons, residual sodium nitrite and TBARS as variables.

## 3. Results

### 3.1. Aw and pH

The Aw and pH of the Larou products of artisanal and industrial origin are shown in Table 1. The Aw values of all samples ranged from 0.662 to 0.859, and the average Aw value of Larou from a traditional farmhouse was lower than that from an industrial source. The lowest Aw value was found in the SY group, while the highest value was detected in the CS group, and no significant differences were observed among the CS, YSH and TRS groups (*p* > 0.05). The Aw values of the YPT and ZJ groups were 0.792 and 0.705, respectively, showing a significant difference with regard to other groups (*p* < 0.05). The average pH of handcrafted Larou products was 5.82, which was lower than that of industrial Larou (pH 6.02). Among them, the pH of the SY group was significantly lower than those of the other Larous (*p* < 0.05). The pH values in the YPT and YSH groups were 5.55 and 6.18, respectively, showing a significant difference (*p* < 0.05). The TRS group of the liquid-smoked Larou exhibited the highest pH value, 6.32, which was significantly higher than those of other Larous (*p* < 0.05). No significant differences were found between the SY group (5.51) and the YPT group (*p* > 0.05).

### 3.2. TBARS and Nitrite Contents

The TBARS represented the secondary products of lipid oxidation (Table 1). The TBARS of Larou in industrial conditions were significantly lower than those of artisanally produced Larou (*p* < 0.05). The TBARS values in SY, CS, ZJ, YPT, YSH and TRS Larou were 1.02, 0.67, 0.62, 0.54, 0.42 and 0.47 mg MDA/kg, respectively, which was significantly different between the two adjacent treatment groups (*p* < 0.05). Residual nitrite contents in the CS and ZJ groups from family-made samples were significantly higher than those of the industrial samples (*p* < 0.05). The residual nitrite contents of all samples were in the range of 1.75 to 3.14 mg/kg, with the lowest value found in the YSH group and the highest in the ZJ group. Among the individual samples, there was no significant difference in residual nitrite contents between the SY group and all three factory-made Larou samples (*p* > 0.05).

### 3.3. Nitrosamines Contents

The NA (NDMA, N-nitrosomethylethylamine (NMEA), NDEA, N-nitrosodi-n-propylamine (NDPA), NDBA, NPIP, NPYP, NMOR and N-nitrosodiphenylamine (NDPhA)) contents of Larou are shown in Table 2. The detection limits of NAs in this study were <0.1 ppm, while NDPhA was not observed in any sample and therefore not included in the table. In the current study, NPYR levels in Larou in artisanal conditions were significantly higher than those in factory-made Larou (*p* < 0.05). The NPYR contents of the SY, ZJ, and CS groups were 2.58, 1.96 and 3.17 μg/kg, respectively. The NPYR contents in the YSH and TRS groups were detected as 1.35 and 0.59 μg/kg, respectively, and in the YPT group not observed, which was significantly lower than all three farmhouse-made Larou samples. The total levels of NAs in industrial Larou were within the range of 3.73 to 7.40 μg/kg. However, among the farmhouse Larou, the total NAs in the ZJ group were detected as 10.78 μg/kg. The highest contents of NMEA (1.56 μg/kg) and NDBA (2.07 μg/kg) were detected in the ZJ group, while NMOR was only detected in the YSH group.

### 3.4. Polycyclic Aromatic Hydrocarbon Analysis

The BaA, Ch, BbF, BkF, BaP, DbA and BgP were determined as main PAHs, while the level of IP in all samples was below the limit of detection (Table 3). The contents of BaP in artisanal Larou products were within the range of 0.25 to 0.78 μg/kg, slightly lower than those of industrial Larou, ranging from 0.30 to 1.87 μg/kg. The contents of PAH4, including BaA, Ch, BbF, and BaP, in three industrial Larou products ranged from 1.62 to 11.92 μg/kg, which was less than the maximum level of 12.00 μg/kg imposed by the current EU regulation no. 835/2011. However, the PAH4 contents in the CS and ZJ groups were detected as 22.48 μg/kg and 19.35 μg/kg, respectively, being significantly higher than those in other samples (*p* < 0.05) and exceeding the maximum limit. The total levels of PAH8 in industrial Larou were with the range of 2.61 to 14.10 μg/kg. The total content of PAH8 in the artisanal Larou group was more than twice that of the industrial Larou in the current study, which was 61.02 μg/kg.

### 3.5. Correlation Analysis among Parameters

**Figure 1:**
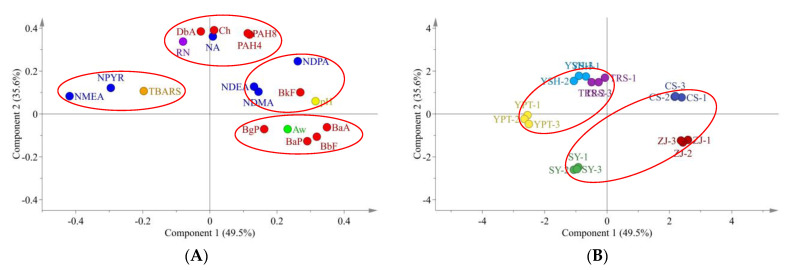
PCA loading plot in the plane of principal component 1 vs. principal component 2 (**A**), and PCA score plot of Larou products from artisanal and industrial origin (**B**) (*n* = 3/group). Abbreviations: Aw, water activity, PAH, polycyclic aromatic hydrocarbon, NA, nitrosamine, RN, residual nitrite, TBARS, thiobarbituric acid reactive substances, NDMA, N−nitrosodimethylamine, NMEA, N−nitrosomethylethylamine, NDEA, N−nitrosodiethylamine, NDPA, N−nitrosodi−n−propylamine, NPYR, N−nitrosopyrrolidine, BaA, benzo(a)anthracene, Ch, chrysene, BbF, benzo(b)fluoranthene, BkF, benzo(k)fluoranthene, BaP, benzo(a)pyrene, DbA dibenzo(a,h)anthracene, BgP, benzo-(g,h,i)perylene. SY, CS and ZJ represent Larou from the farms of Shaoyang City, Changsha City and Zhangjiajie City, in Hunan Province, respectively. YPT, YSH and TRS are from factories in Hunan Province.

The PCA analysis and correlations among pH, Aw, TBARS, residual nitrite, NAs and PAHs from all Larou samples are shown in Figure 1 and Table 4 and Table 5, respectively. The two principal components accounted for 85.1% of the total variation (49.5% and 35.6%, respectively) in the PCA model. The first principal component (PC1) was related with pH, Aw, some nitrosamines (NDPA, NDEA and NDMA) and PAHs, except for DbA. PC2 was positively related with residual nitrite and contents of PAH and NA, which were clustered in the same group. Similarly, there was a strong correlation between the total NAs and residual nitrites in all Larou products (τ = 0.805, *p* < 0.01), among which a positive coefficient index between residual nitrites and the amount of NDPA (τ = 0.626, *p* < 0.05), NDBA (τ = 0.913, *p* < 0.05) and NPYR (τ = 0.596, *p* < 0.05) were observed (Table 4). The NMEA, NPYR and TBARS were adjacently clustered in a group of PCA plot, indicating a positive relationship. Moreover, significant positive correlations were also found between TBARS values and NDBA (τ = 0.970, *p* < 0.01), NPIP (0.869, *p* < 0.05) and NPYR (τ = 0.590, *p* < 0.05). It was found that pH was positively correlated with NDEA (τ = 0.671, *p* < 0.01) and NDPA (τ = 0.594, *p* < 0.05), but negatively correlated with NMEA (τ = −0.850, *p* < 0.01), NDBA (τ = −0.961, *p* < 0.01), NPIP (τ = −0.843, *p* < 0.05) and NPYR (τ = −0.789, *p* < 0.01). Aw showed a negative correlation with NMEA (τ = −0.825, *p* < 0.01), NDBA (τ = −0.961, *p* < 0.01) and NPYR (τ = −0.746, *p* < 0.01).

The distributions of Larou samples from artisanal and industrial sources in the PCA score plot was shown in Figure 1B. Factory-made Larou samples (YPT, YSH and TRS) seemed to be grouped together, and significantly different from family-made Larou. Further, the CS, SY and ZJ samples of family-made Larou were spread over the first, third and fourth quadrants in the PCA plot.

As shown in Table 5, a positive correlation between PAH8 and residual nitrites (τ = 0.692, *p* < 0.01) was observed, which is consistent with a close distribution in the PCA plot (Figure 1A). Significant positive correlations were found between residual nitrites and Ch (τ = 0.766, *p* < 0.01) and DbA (τ = 0.907, *p* < 0.01). It was noticed that BaA was positively correlated to the Aw value (τ = 0.658, *p* < 0.01). Significant positive correlations were found between pH values and BaA (τ = 0.807, *p* < 0.01), BbF (τ = 0.601, *p* < 0.01), BkF (τ = 0.510, *p* < 0.05) and BaP (τ = 0.475, *p* < 0.05).

## 4. Discussion

The difference of Aw value might be related to the size and thickness of Larou and the surface area of Larou, which affected the dehydration during processing [10]. The lower Aw values in the farmhouse-made Larou of the SY and ZJ groups may be due to the long smoking process—as much as 35 d, compared to 12 h of industrial smoking. In addition, the uniformly automatic smoking roaster in the factory made the surface of Larou absorb substances such as phenolic resin to form a film, thus slowing down the evaporation of the surface moisture [1]. It was found that the Aw of all Larou samples were lower than 0.9, which essentially inhibited most pathogenic bacteria and contributed to the extended shelf-life under room temperature [21]. Guo et al. [22] reported that the pH of 15 traditional Chinese pork bacon samples from Hunan also ranged from 5.38 to 6.38, which is consistent with our findings. The salting ingredients, the phenolic compounds adsorbed during smoking and meat proteolysis might contribute to the pH variation among Larou samples. For example, Wang et al. [1] reported that the pH values of bacon decreased with the addition of polyphenols during dry-ripening and storage.

Family-made Larou samples were made and stored to be directly exposed to oxygen and sunlight, and the lipids were susceptible to oxidation. In contrast, factory Larous were vacuum-packed and stored under refrigeration, which prevented them from interacting with oxygen, and thus retarded the development of lipid oxidation [23]. Furthermore, some phenolic compounds in the smoke which gave the desirable flavor acted as antioxidative compounds for the smoked product [2]. Wang et al. [9] confirmed that the addition of the plant polyphenol compounds and antioxidant ingredients of spices exhibited an inhibition effect against lipid oxidation in the bacon. The nitrite residue of Larou in industrial conditions was lower than that of artisanal Larou. Herrmann, Duedahl-Olesen and Granby [8] found that the mean contents of nitrite residue in salami, sausages, ham and bacon from the Danish and Belgian markets were about 6.0 and 4.0 mg/kg, respectively. Our results showed that the nitrite residues of all Larous were far less than 30 mg/kg, which was the limit standard of China for nitrite in meat product [24], suggesting the safety of residual nitrite in Larou destined for consumption. As the meat was below pH 6.0 or less, the nitrite can be transformed into nitrous acid or nitric oxide, which further reacts with polyphenols to form nitroso compounds [25].

The IARC considered NDMA and NDEA as probably carcinogenic for humans based on epidemiological studies and animal experiments. The contents of NDMA in all Larou products were lower than the tolerance limit of 3 μg/kg established by National Standard of the People’s Republic of China [4]. During meat processing, the degradation of proteins and lipids may generate the precursors of nitrosamines. For example, NDMA can be formed by the combination of a deprotonated amine from lecithin, glycine or sarcosine with a nitroso group, while NDEA can be formed from alanine [26]. Moreover, if artisanal Larou is stored hanging in the air, this may increase the NA contents, as demonstrated during the prolonged storage of high-oxygen-packaged pork. The lipid and protein oxidation of the meat produced degraded compounds which may function as the necessary amine precursors [27]. It was proposed that the degradation of sarcoplasmic proteins and the formation of carbonyls contributed to an increased NDEA and NDMA formation when the aged pork meat was treated with nitrite [28].

The levels of NAs may increase as a result of heat treatment, especially NDMA and NPYR [12]. It was suggested that a high smoking temperature and a long smoking time in traditional processing would lead to a significant increase in NPYR content. Moreover, NPYR may originate from pyrrolidine- and piperidine-containing spices like black pepper and paprika. However, the use of such premixes was strongly discouraged [29]. Nevertheless, in a recent study, high amounts of piperine and piperidine in the spices which were used in the premixes with nitrite showed no necessary association with the formation of NPIP [30]. This is consistent with our finding that NPIP was not detected in the ZJ and YSH groups, which used spices as additives in salting process. It was suggested that the extent of NA formation may be related to the storage conditions of the premixes and the addition of the spices. The pork processed by artisanal technology may contain rich sources of secondary and tertiary amines, as the crude salt used to cure the ingredients included nitrate and possibly nitrite [8]. The main sources of nitrosyl donors were not only sodium nitrite from the curing procedure but also gaseous nitrogen oxides in the smoking process. The smoking liquid may contain increased concentrations of NOx compounds such as NO and NO_2_, acting as precursors of nitrosating agents. On the other hand, combustion smoke may contain NAs from burning wood, mainly NDMA, and to a lesser extent NPIP and NMOR [31]. As far as the current study is concerned, Larou in industrialized conditions had relatively less accumulation of NAs.

Especially, BaP is used as a marker for the occurrence of carcinogenic PAHs in food due to its higher associations with cancer in humans. Kafouris, Koukkidou, Christou, Hadjigeorgiou and Yiannopoulos [32] revealed that the level of BaP in traditional smoked bacon from Cyprus was 1.20 μg/kg. The values obtained for BaP in all Larou products were lower than the maximum permissible limit (5.00 μg/kg) in China. The levels of BaP in Larou products in the current study seemed to be considered acceptable to consumers without health concerns. However, according to the European Food Safety Authority (EFSA), Bap is not sufficient for indicating the presence of PAHs in food, and the most appropriate candidate is PAH4, consisting of BaA, Ch, BbF and BaP. Moreover, the amount of total PAH compounds (PAH8) by the sum of BaA, Ch, BbF, BkF, BaP, DbA, BgP and IP was also regarded as an indicator for the occurrence of PAHs in food, but it is also still being discussed by the EFSA committee [33].

Researchers stated that PAH formation was highly dependent on the heating temperature and the time in both dry and wet conditions [34]. The smoking time in handcrafted Larou products in this study was much longer than that of industrial Larou, which seemed to explain the high contents of PAHs in artisanal Larou. Another factor affecting the level of the PAHs of Larou is the circulation of smoke in the smoking chamber. Generally, traditional smoking houses performed no circulation of the smoke and were unable to smoke sufficiently and evenly for Larou. This would cause a direct exposure of pork meat to the smoke, resulting in increased concentrations of PAHs compared to the products smoked by an indirect approach [35]. Zachara, Gałkowska and Juszczak [36] had found that the sausages and hams which were hot-smoked using the traditional technique, i.e., directly over the fireplace, contained more polycyclic aromatic hydrocarbons than similar products smoked using the industrial technique. Djinovic, Popovic and Jira [37] also found that the PAH4 contents were significantly higher in the samples of hams smoked with traditional methods than with industrial ones, which is consistent with our finding that the content of PAH in Larou (YPT) was the lowest. The type of smoked wood plays a significant role in the contamination of the PAH contents in smoked meat products. Meat smoked with softwood was reported to have higher PAH levels than that of hardwood, probably resulting from the chemical compounds in the wood and the different natures of the wood types [38].

The accumulation of NA was closely related with residual nitrites, which are commonly known as precursors for the formation of carcinogenic N-nitrosamines. Studies have indicated that there is a positive but not necessarily linear correlation between the amount of added nitrite and the amount of formed NA in cured pork meat products [12,39,40]. Besides, earlier reports suggested that the oxidation of the adipose tissue would result in the formation of malondialdehyde, a secondary product of lipid oxidation which may promote NA formation [41]. It was suggested that the addition of natural plant antioxidants was able to delay lipid oxidation and reduce the accumulation of NA during the production of Larou [9,42].

There is a correlation between the physical and chemical properties and the level of NAs in Larou. The risk of NPIP formation from piperidine in dry fermented sausages was estimated by using a protein-based liquid system, which found a significant increase in NPIP levels at lower pH [43]. In addition, Kaban, Polat, Sallan and Kaya [44] found that the accumulation of NPIP and NPYR and the pH value of 10 different brands of heat-treated semi-dry fermented sausages on the Turkish market were also negatively correlated. Moreover, fermented sausages with added starter culture showed higher NPIP and NPYR levels than samples without starter culture due to the lower pH value [45]. In addition, Sallan et al. [45] also reported that NDMA was not affected by the pH value, which is consistent with our finding that no significant correlation between NDMA and the pH value was found. Kaban, Polat, Sallan and Kaya [44] also proved that NPYR was negatively correlated with Aw through a PCA analysis. Recent research showed that the count of Enterobacteriaceae and Pseudomonadaceae, which were potential producers of biogenic amines (BAs), was significantly correlated with Aw during the ripening period of dry-smoked sausage from Serbia [46]. Even though Aw values gradually decreased during the ripening process, the gradually accumulated BAs like butylamine, spermine and spermidine can form carcinogenic NAs through a nitration reaction [47]. This seems to explain the negative correlation between Aw values and NAs.

It is interesting to note that a positive correlation between PAH8 and residual nitrites was found in this study. This indicates that the smoking procedure of Larou accompanied with thermal treatments might cause a synchronous formation of nitrite and PAHs in meat products [48]. Incomplete wood combustion during the smoking process can produce considerable amounts of PAHs, which were adsorbed on the surface of the Larou. Meanwhile, the moisture content of Larou decreased, accompanied by an increase in the nitrite content due to the dehydrating properties of smoke and the continuous heat treatment. Deng et al. [14] found that the residual nitrite content level increased (from unheated control to 150 °C) in the bacon samples with the same sodium nitrite levels. Although the specific mechanism of the influence between PAHs and residual nitrite was unclear, the highest PAH values of bacon were observed in the curing process compared to other processes [49]. The maximum permissible amount of nitrite for cured meat products is 150 mg/kg, according to the National Standard of the People’s Republic of China [24], and thus it is still recommended to reduce the addition of nitrite in artisanal and industrial Larou for health risk reduction.

Variations of pH could affect the generation of PAHs in meat products, which might be attributed to the influence of pH on the Maillard reactions. These nonenzymatic reactions occur between sugars and proteins during heating, and the intermediate products, such as the proline Amadori compound, can further generate PAHs such as pyrene, BaA and BaP [50]. Wongmaneepratip and Vangnai [51] found that a slight increase in pH could significantly increase the PAH content in grilled chicken. Li et al. [15] reported that the formation of PAH was significantly correlated with pH (τ = 0.962, *p* < 0.01) in bacon, which is consistent with our findings. The pH variation induced by the characteristic of the substitute salt played a vital role in this mechanism, whereby the Maillard reactions impact the generation of PAHs. Therefore, in order to reduce the production of harmful compounds in Larou, the contents of added salts and pickling ingredients may be reconsidered. However, due to the limitations of the obtained data in the current study (*n* = 3/group), more replicates of Larou from artisanal and industrial origin need to be included to robustly predict the correlations in future studies.

According to the PCA score plot of Larou products from artisanal and industrial origin, it is suggested that the Larou from the industrial sources is similar in safety attributes, which are more stable than those of Larou processed in an artisanal manner. However, the distribution of family-made Larou showed a greater randomness, indicating variable safety and physicochemical attributes. This was in accord with the non-standardized processing regulation in farmhouse-made Larou, producing the random effect on its characteristic and safety concerns. Taken together, in terms of safety attributes and including residual nitrite, NA and PAH, the factory-made Larou is encouraged due to the standardized operation process to guarantee minimal health risks.

## 5. Conclusions

This study mainly compared the contents of nitrosamines and polycyclic aromatic hydrocarbons in Larou from artisanal and industrial sources. All Larou samples presented minimal safety concerns, since nitrosamines and polycyclic aromatic hydrocarbons were within the acceptable ranges stipulated by the National Standard of the People’s Republic of China. Nevertheless, two farmhouse-made Larou products (CS and ZJ) possessed a health risk because of the high level of PAH4 (>12 μg/kg), and the Larou samples in the ZJ group exceeded the 10 μg/kg for NAs. Hence, the smoking, pickling and storage process of Larou products in artisanal processing should be reconsidered. The total amount of NAs and PAHs in industrialized Larou was lower than that of farm-type Larou. Meanwhile, the principal component analysis showed that the safety of factory-made Larou was more stable than that of farmhouse-made Larou. It is concluded that factory-made Larou is more appropriate for consumption, with minimal health concerns. Further work is required to control the Larou processing parameters and the standard production conditions for farmhouse production.

## Figures and Tables

**Table 1:** Aw, pH, residual nitrite content and TBARS values in Larou of artisanal and industrial origin.

Index	Artisanal Origin			Industrial Origin		
	SY	CS	ZJ	YPT	YSH	TRS
Aw	0.662 ± 0.002 ^d^	0.859 ± 0.002 ^a^	0.705 ± 0.003 ^c^	0.792 ± 0.004 ^b^	0.855 ± 0.014 ^a^	0.846 ± 0.011 ^a^
pH	5.51 ± 0.03 ^d^	6.21 ± 0.01 ^b^	5.74 ± 0.02 ^c^	5.55 ± 0.13 ^d^	6.18 ± 0.03 ^b^	6.32 ± 0.05 ^a^
TBARS (mg MDA/kg)	1.02 ± 0.05 ^a^	0.67 ± 0.03 ^b^	0.62 ± 0.02 ^c^	0.54 ± 0.03 ^d^	0.42 ± 0.02 ^f^	0.47 ± 0.04 ^e^
Residual nitrites (mg/kg)	1.79 ± 0.21 ^c^	2.70 ± 0.04 ^b^	3.14 ± 0.26 ^a^	2.03 ± 0.17 ^c^	1.75 ± 0.05 ^c^	1.88 ± 0.06 ^c^

SY, CS and ZJ represent Larou from the farms of Shaoyang City, Changsha City and Zhangjiajie City, in Hunan Province, respectively. YPT, YSH and TRS are from factories in Hunan Province. The symbols represent the means ± standard deviation obtained from three independent experiments. Means with different letters in the same row are significantly different (*p* < 0.05, *n* = 3/group).

**Table 2:** Content of nitrosamines (μg/kg) in Larou of artisanal and industrial origin.

Compounds	Artisanal Origin			Industrial Origin		
	SY	CS	ZJ	YPT	YSH	TRS
NDMA	1.64 ± 0.18 ^a^	1.58 ± 0.13 ^a^	0.90 ± 0.10 ^b^	0.60 ± 0.05 ^c^	1.43 ± 0.21 ^a^	1.11 ± 0.28 ^b^
NMEA	1.40 ± 0.09 ^a^	1.05 ± 0.15 ^b^	1.56 ± 0.13 ^a^	nd	nd	0.74 ± 0.16 ^c^
NDEA	nd	1.32 ± 0.13 ^b^	1.01 ± 0.13 ^c^	0.68 ± 0.11 ^d^	0.78 ± 0.06 ^d^	1.70 ± 0.15 ^a^
NDPA	nd	2.25 ± 0.27 ^a^	2.08 ± 0.25 ^a^	0.20 ± 0.09 ^c^	nd	1.59 ± 0.46 ^b^
NDBA	nd	nd	2.07 ± 0.67 ^a^	nd	nd	0.27 ± 0.03 ^b^
NPIP	nd	nd	nd	2.25 ± 0.37 ^a^	nd	1.39 ± 0.53 ^b^
NPYR	2.58 ± 0.27 ^b^	1.96 ± 0.29 ^c^	3.17 ± 0.76 ^a^	nd	1.35 ± 0.29 ^d^	0.59 ± 0.24 ^e^
NMOR	nd	nd	nd	nd	0.51 ± 0.21 ^a^	nd
∑NA8	5.62 ± 0.54 ^d^	8.17 ± 0.17 ^b^	10.78 ± 0.24 ^a^	3.73 ± 0.52 ^e^	4.07 ± 0.77 ^e^	7.40 ± 0.93 ^c^

SY, CS and ZJ represent Larou from the farms of Shaoyang City, Changsha City and Zhangjiajie City, in Hunan Province, respectively. YPT, YSH and TRS are from factories in Hunan Province. NDMA, NMEA, NDEA, NDPA, NDBA, NPIP, NPYR and NMOR indicate N-nitrosodimethylamine, N-nitrosomethylethylamine, N-nitrosodiethylamine, N-nitrosodi-n-propylamine, N-nitrosodi-n-butylamide, N-nitrosopiperidine, N-nitrosopyrrolidine and N-nitrosomorpholine, respectively. The symbols represent the means ± standard deviation obtained from three independent experiments. Means with different letters in the same row are significantly different (*p* < 0.05, *n* = 3/group). “nd” indicates “Not detected”.

**Table 3:** Content of polycyclic aromatic hydrocarbons (μg/kg) in Larou of artisanal and industrial origin.

Compounds	Artisanal Origin			Industrial Origin		
	SY	CS	ZJ	YPT	YSH	TRS
BaA	0.50 ± 0.11 ^de^	1.17 ± 0.24 ^c^	0.71 ± 0.13 ^d^	0.20 ± 0.02 ^e^	2.73 ± 0.39 ^a^	1.97 ± 0.26 ^b^
Ch	9.14 ± 0.70 ^c^	20.62 ± 0.97 ^a^	17.19 ± 1.07 ^b^	0.95 ± 0.01 ^f^	4.95 ± 0.39 ^e^	6.33 ± 0.20 ^d^
BbF	0.42 ± 0.12 ^d^	0.44 ± 0.01 ^d^	0.68 ± 0.03 ^c^	0.16 ± 0.01 ^e^	2.36 ± 0.20 ^a^	1.23 ± 0.03 ^b^
BkF	0.32 ± 0.02 ^b^	0.28 ± 0.01 ^c^	0.51 ± 0.03 ^a^	0.11 ± 0.01 ^d^	0.52 ± 0.02 ^a^	0.49 ± 0.01 ^a^
BaP	0.59 ± 0.06 ^d^	0.25 ± 0.02 ^e^	0.78 ± 0.07 ^c^	0.30 ± 0.06 ^e^	1.87 ± 0.11 ^a^	1.11 ± 0.07 ^b^
DbA	1.02 ± 0.07 ^c^	2.22 ± 0.04 ^b^	2.74 ± 0.08 ^a^	0.49 ± 0.01 ^f^	0.80 ± 0.20 ^d^	0.68 ± 0.14 ^de^
BgP	0.55 ± 0.04 ^c^	0.34 ± 0.01 ^e^	0.59 ± 0.01 ^b^	0.39 ± 0.01 ^d^	0.86 ± 0.02 ^a^	0.38 ± 0.01 ^d^
∑PAH4	10.64 ^c^	22.48 ^a^	19.35 ^b^	1.62 ^d^	11.92 ^c^	10.64 ^c^
∑PAH8	12.53 ^d^	25.31 ^a^	23.18 ^b^	2.61 ^e^	14.10 ^c^	12.18 ^d^

SY, CS and ZJ represent Larou from the farms of Shaoyang City, Changsha City and Zhangjiajie City, in Hunan Province, respectively. YPT, YSH and TRS are from factories in Hunan Province. BaA, Ch, BbF, BkF, BaP, DbA and BgP indicate benzo(a)anthracene, chrysene, benzo(b)fluoranthene, benzo(k)fluoranthene, benzo(a)pyrene, dibenzo(a,h)anthracene and benzo-(g,h,i)perylene, respectively. The symbols represent the means ± standard deviation obtained from three independent experiments. Means with different letters in the same row are significantly different (*p* < 0.05, *n* = 3/group).

**Table 4:** Correlation between Aw, pH, TBARS, residual nitrites, NDMA, NMEA, NDEA, NDPA, NDBA, NPIP, NPYR, NMOR and NA in Larou of artisanal and industrial origin.

	Aw	pH	TBARS	RN	NDMA	NMEA	NDEA	NDPA	NDBA	NPIP	NPYR	NMOR	NA
Aw	1	0.819 **	−0.766 **	−0.161	0.027	−0.825 **	0.319	0.038	−0.961 **	−0.727	−0.746 **	0.982	−0.204
pH		1	−0.592 **	−0.046	0.305	−0.850 **	0.671 **	0.594 *	−0.961 **	−0.843 *	−0.789 **	0.364	0.171
TBARS			1	0.029	0.452	0.570	0.081	0.537	0.970 **	0.869 *	0.590 *	0.634	0.110
RN				1	−0.201	0.437	0.038	0.626 *	0.913 *	0.410	0.596 *	−0.500	0.805 **
NDMA					1	−0.063	0.342	0.694 *	−0.545	−0.973 **	−0.059	1.000 **	0.000
NMEA						1	−0.981 **	0.481	0.972 **	1.000 **	0.925 **	-	0.338
NDEA							1	0.552	−0.973 **	−0.867 *	−0.472	1.000 **	0.481
NDPA								1	0.620	−0.696	0.644	-	0.890 **
NDBA									1	−1.000 **	0.860 *	-	0.913 *
NPIP										1	1.000 **	-	−0.711
NPYR											1	1.000 **	0.487
NMOR												1	1.000 **
NA													1

*: Pearson correlation coefficient was at significant levels of *p* < 0.05, *n* = 3/group. **: Pearson correlation coefficient was at significant levels of *p* < 0.01, *n* = 3/group. -: Pearson correlation coefficient cannot be counted. NDMA, NMEA, NDEA, NDPA, NDBA, NPIP, NPYR, NMOR and NA indicate N-nitrosodimethylamine, N-nitrosomethylethylamine, N-nitrosodiethylamine, N-nitrosodi-n-propylamine, N-nitrosodi-n-butylamide, N-nitrosopiperidine, N-nitrosopyrrolidine, N-nitrosomorpholine and Nitrosamines, respectively.

**Table 5:** Correlation between Aw, pH, TBARS, residual nitrites, BaA, Ch, BbF, BkF, BaP, DbA, BgP, NA, PAH4 and PAH8 in Larou of artisanal and industrial origin.

	Aw	pH	TBARS	RN	BaA	Ch	BbF	BkF	BaP	DbA	BgP	NA	PAH4	PAH8
Aw	1	0.819 **	−0.766 **	−0.161	0.658 **	−0.098	0.468	0.068	0.304	−0.235	−0.103	−0.204	0.064	0.030
pH		1	−0.592 **	−0.046	0.807 **	0.197	0.601 **	0.510 *	0.475 *	0.023	0.011	0.171	0.414	0.383
TBARS			1	0.029	−0.586 *	0.305	−0.564 *	−0.292	−0.504 *	0.194	−0.145	0.110	0.131	0.129
RN				1	−0.331	0.766 **	−0.381	−0.060	−0.397	0.907 **	−0.223	0.805 **	0.665 **	0.692 **
BaA					1	−0.124	0.922 **	0.680 **	0.863 **	−0.219	0.510 *	−0.119	0.179	0.161
Ch						1	−0.256	0.180	−0.338	0.921 **	−0.190	0.811 **	0.951 **	0.952 **
BbF							1	0.737 **	0.970 **	−0.255	0.757 **	−0.218	0.050	0.049
BkF								1	0.780 **	0.226	0.582 *	0.406	0.420	0.434
BaP									1	−0.287	0.799 **	−0.207	−0.042	−0.035
DbA										1	−0.055	0.848 **	0.862 **	0.885 **
BgP											1	−0.223	−0.022	0.048
NA												1	0.774 **	0.788 **
PAH4													1	0.998 **
PAH8														1

*: Pearson correlation coefficient was at significant levels of *p* < 0.05, *n* = 3/group. **: Pearson correlation coefficient was at significant levels of *p* < 0.01, *n* = 3/group. BaA, Ch, BbF, BkF, BaP, DbA, BgP and NA indicate benzo(a)anthracene, chrysene, benzo(b)fluoranthene, benzo(k)fluoranthene, benzo(a)pyrene, dibenzo(a,h)anthracene, benzo-(g,h,i)perylene and Nitrosamines, respectively. PAH4 including BaA, Ch, BbF, and BaP. PAH8 including PAH4, BkF, BaP, DbA and IP.

## Data Availability

The data presented in this study are available in the article.
